# Age‐Dependent Variation in Blood Biopterin Peaks Following Oral Tetrahydrobiopterin Administration in Phenylketonuria

**DOI:** 10.1002/jimd.70154

**Published:** 2026-02-08

**Authors:** Kana Kitayama, Tomoko Sakaguchi, Noriko Nakano, Daijiro Kabata, Haruo Shintaku, Takashi Hamazaki

**Affiliations:** ^1^ Department of Pediatrics Osaka Metropolitan University Graduate School of Medicine Osaka Japan; ^2^ Center for Mathematical and Data Science Kobe University Kobe Japan; ^3^ Donated Course “Human Resource Development in Regional Perinatal Neonatal Care” Osaka Metropolitan University Graduate School of Medicine Osaka Japan

**Keywords:** BH_4_ responsiveness, neonatal, phenylketonuria, sapropterin, tetrahydrobiopterin bioavailability, tetrahydrobiopterin loading test

## Abstract

The correct diagnosis of tetrahydrobiopterin (BH_4_, sapropterin dihydrochloride)‐responsive phenylketonuria (PKU) and treatment with BH_4_ are important for prognosis and quality of life. We examined whether age affects biopterin bioavailability following oral BH_4_ administration in PKU and whether this influences BH_4_ responsiveness. A retrospective analysis was conducted in 255 Japanese PKU patients who underwent a 24‐h BH_4_ loading test (mostly ≤ 2 months old) and/or a 1‐week test (all ≥ 5 months) between 2008 and 2023. Correlations were evaluated among age, peak blood biopterin, and the phenylalanine (Phe) reduction rate. In the 24‐h test, analyses of day‐of‐age versus peak biopterin after oral BH_4_ showed that peak levels were highest during the early neonatal period and declined significantly with age (*p* = 0.008). In the 1‐week test, peak levels increased during school age through adolescence (6–19 years) (*p* = 0.001), with no material age trend in adults (≥ 20 years). In the 24‐h test, peak biopterin correlated positively with the Phe reduction rate (*p* = 0.029), and baseline Phe correlated negatively with peak biopterin (*p* = 0.001). These findings indicate that BH_4_ loading tests performed in infants and preschool‐aged children (1 month–5 years)—in whom biopterin peaks tend to be low—or in patients with high baseline Phe levels may yield suppressed biopterin peaks and false‐negative results. Reassessment of BH_4_ responsiveness and age‐appropriate dose adjustment should be considered when necessary.

## 1 Introduction

Phenylketonuria (PKU; OMIM #261600) is an autosomal recessive metabolic disorder caused by pathogenic variants in the phenylalanine hydroxylase (PAH; EC 1.14.16.1; OMIM 612349) gene [1]. Without treatment, elevated blood phenylalanine (Phe) levels lead to neurodevelopmental delay, seizures, and behavioral disturbances [2]. The standard treatment consists of dietary management with Phe‐free amino acid supplements combined with limited amounts of natural protein to maintain appropriate blood Phe levels; however, a subset of patients can be treated with tetrahydrobiopterin (BH_4_, sapropterin dihydrochloride). These patients are referred to as having BH_4_‐responsive PKU, and because oral BH_4_ administration can alleviate the strict dietary restrictions, accurate diagnosis of BH_4_‐responsive PKU is therefore important for improving patients' quality of life [1, 3]. The BH_4_ loading test is commonly used to assess BH_4_ responsiveness [4]. In Japan, a 24‐h loading test is typically performed in the early neonatal period following detection of hyperphenylalaninemia through newborn screening. Patients who do not respond may undergo a 1‐week BH_4_ trial beginning at approximately 5 months of age, when weaning starts, or later in childhood or adulthood.

An increase in the intracellular concentration of BH_4_ lowers blood Phe levels in a dose‐dependent manner. In mouse models, increased intracellular BH_4_ significantly enhances Phe metabolism [5], and similar dose‐dependent effects have been reported in genetically homogeneous human cohorts with BH_4_‐responsive PKU [6]. BH_4_ bioavailability is known to vary substantially both between individuals and within the same individual. Population pharmacokinetic and clinical data indicate that age, body weight and intestinal handling of sapropterin modulate systemic exposure, and that there is marked inter‐ and intra‐individual variability in C_max and AUC even at the same mg/kg dose [7]. Moreover, timing‐related factors—in particular the interval between BH_4_ administration and blood sampling, and the timing and fat content of the preceding meal, which can increase sapropterin C_max by approximately 40%–80% compared with fasting [8]—as well as the dosing schedule (e.g., once‐daily versus divided dosing) [9] and possible diurnal variation in circulating pterin levels [10], can all influence the measured biopterin peak and thereby the apparent outcome of BH_4_ loading tests.

In our previous case report, repeated 24‐h BH_4_ loading tests in the same patients demonstrated that peak total biopterin concentrations and the Phe reduction rate decreased in parallel over time. In a patient with the *PAH* p.Arg241Cys/Arg111Ter genotype, peak total biopterin levels declined from 747 nM on Day 20 to 327 nM on Day 55, with corresponding Phe reduction rates of 39% and 32%. In another patient with the *PAH* p.Pro407Ser/Arg158Trp genotype, peak biopterin levels were 612, 297, and 178 nM at Day 30, Day 55, and 19 months of age, respectively, with associated Phe reduction rates of 54%, 16%, and 4% [11]. Based on these findings, we hypothesize that BH_4_ bioavailability declines rapidly after the early neonatal period and that this decline affects diagnosis of BH_4_ responsiveness, as measured by the rate of Phe reduction.

In this study, we estimated the bioavailability of BH_4_ and its impact on the Phe reduction rate using data from 24‐h and 1‐week BH_4_ loading tests. We analyzed the relationships between age and peak biopterin levels, as well as between peak biopterin levels and the Phe reduction rate. To date, there have been no studies investigating BH_4_ absorption in human populations that include a large number of neonates. In this study, we analyzed BH_4_ loading test data from 255 PKU patients (oldest participant: 51 years old) that included 111 neonates younger than 30 days (youngest participant: 8 days old).

A retrospective analysis was conducted using BH_4_ loading test data from patients at Osaka Metropolitan University, the reference center in Japan equipped to perform pterin analysis for PKU.

## 2 Methods

### 2.1 Study Design and Participants

The study included 255 Japanese patients (94 males, 156 females, and 5 of unknown sex) who underwent a 24‐h and/or a 1‐week BH_4_ loading test at Osaka Metropolitan University Graduate School of Medicine (Osaka, Japan) between January 2008 and June 2023 (24‐h, *n* = 170; 1‐week, *n* = 117; overlap, *n* = 32).

All patients had plasma Phe concentrations > 360 μM at diagnosis or during follow‐up, and were biochemically diagnosed with PKU after exclusion of BH_4_ deficiency based on blood and urinary levels of neopterin, biopterin, and 7‐biopterin, as well as dihydropteridine reductase activity measured in dried blood spots.

We retrospectively reviewed the medical records to analyze the results of BH_4_ loading tests and clinical features.

### 2.2 Protocols

#### 2.2.1 Twenty‐Four‐Hour BH_4_ Loading Test

**FIGURE 1:**
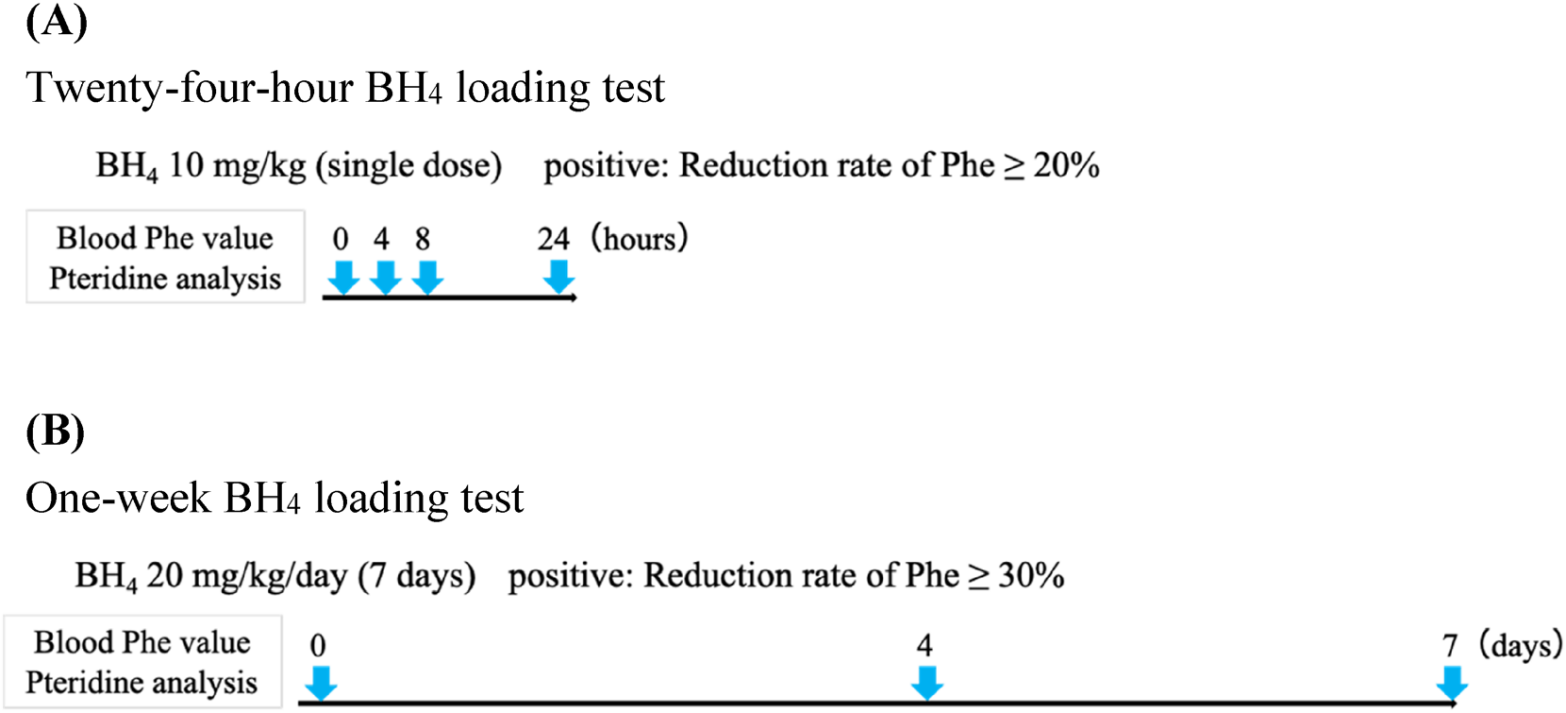
Overview of the BH_4_ loading tests. (A) Twenty‐four‐hour BH_4_ loading test. Patients received 10 mg/kg BH_4_ orally, and blood samples were taken before and at 4, 8, and 24 h after dosing to determine phenylalanine (Phe), total neopterin, and total biopterin levels. Subjects were diagnosed with BH_4_ rapid‐response Phe‐hydroxylase deficiency if they had a maximum Phe decrease of ≥ 20% compared to baseline within 24 h of study entry. (B) One‐week BH_4_ loading test. Patients received 20 mg/kg/day BH_4_ for 1 week orally, and blood samples were taken before and at 4 and 7 days after dosing to determine Phe, total neopterin, and total biopterin levels. Subjects were diagnosed with BH_4_ slow‐response Phe‐hydroxylase deficiency if they had a maximum Phe decrease of ≥ 30% compared to baseline within 1 week of study entry.

The 24‐h BH_4_ loading test was performed when plasma Phe concentrations exceeded 360 μM (Figure 1A). Patients received an oral dose of 10 mg/kg BH_4_, and blood samples were collected at baseline, and at 4, 8, and 24 h post‐administration. Plasma levels of Phe, total neopterin, and total biopterin were measured. Patients were diagnosed with BH_4_ rapid‐response PAH deficiency if their Phe reduction exceeded 20%. In this study, “peak total biopterin concentration” was defined as the maximum value measured among the three post‐administration time points (4, 8, and 24 h).

#### 2.2.2 One‐Week BH_4_ Administration Test

Patients were orally administered 20 mg/kg BH_4_ once daily for 7 consecutive days (Figure 1B). Because this test was conducted in patients who had already initiated treatment, they were instructed to consume a normal diet from 3 days prior to the test until its completion. Blood samples were collected before the first dose, on Day 4, and on Day 7, and plasma levels of Phe, neopterin, and total biopterin were measured. BH_4_ slow‐response PAH deficiency was diagnosed if the maximum Phe reduction from baseline was at least 30% on at least one of Day 4 or 7. The higher value of total biopterin measured on either Day 4 or 7 was designated as the “peak total biopterin concentration” for this test.

### 2.3 Biochemical Analyses

Plasma Phe concentrations were measured using an automated amino acid analyzer (L‐8800; Hitachi, Tokyo, Japan). For pteridine analysis, 1 mL of whole blood was collected in an EDTA tube protected from light and containing 1 mg of ascorbic acid for stabilization. After centrifugation, the plasma supernatant was immediately frozen and stored at −20°C until analysis. Samples were deproteinized immediately after thawing and oxidized with iodine under acidic conditions for at least 3 h at room temperature, and reduced with ascorbic acid before being subjected to HPLC analysis. The details of these procedures have been described previously [12]. Total biopterin concentrations (the sum of BH_4_, qBH2, BH2, and biopterin) were measured via high‐performance liquid chromatography (LC‐10; Shimazu, Kyoto, Japan) with fluorimetric detection.

Dihydropteridine reductase activity was measured in dried blood spot specimens collected on No. 545 filter paper (Toyo Roshi Kaisha Ltd., Tokyo, Japan), as described previously [13].

### 2.4 Statistical Analysis

We modeled associations using ordinary least‐squares regression with restricted cubic splines. All spline terms used 3 knots.

In addition to the primary regression analyses, we stratified the 24‐h and the 1‐week BH_4_ loading tests by age and performed linear regressions within strata to explore the age ranges in which the association between age and peak total biopterin concentration was significant. We stratified the 24‐h BH_4_ loading test into < 30 and ≥ 30 days, and the BH_4_ 1‐week loading test into < 6, 6–19, and ≥ 20 years. In each stratum of both tests, we fitted linear regression models with peak blood biopterin concentration as the outcome and age as the main predictor (day‐of‐age for the 24‐h test; years for the 1‐week test), adjusting only for baseline biopterin. Residuals were checked using histograms, heteroscedasticity was assessed with the Breusch–Pagan test, and HC3 robust standard errors were reported as a sensitivity analysis. *p*‐values for the age effect were adjusted across strata using the Benjamini–Hochberg procedure. Results are reported as slopes (24‐h: nM/day; 1‐week: nM/year) with 95% confidence intervals, Benjamini–Hochberg‐adjusted *q*‐values, adjusted *R* ^2^, and the sample size in each stratum.

For nonlinear regression analyses, selected independent and dependent variables were natural log–transformed to meet the assumption of normal error distribution. The regression models were adjusted for covariates including age, sex, baseline Phe concentration, and baseline total neopterin and biopterin levels, and the effect of each covariate on the outcome was also evaluated individually. All statistical tests were two‐sided with a significance level set at 5%. Statistical analyses were performed using R (https://www.r‐project.org/foundation/, accessed on 29 March 2023; https://cran.r‐project.org/, accessed on 29 March 2023).

This study was approved by the Institutional Review Board of the Graduate School of Medicine, Osaka Metropolitan University (approval no. 2023‐110).

## 3 Results

Out of a total of 255 patients who underwent the BH_4_ loading test, 170 underwent the 24‐h BH_4_ loading test, and 117 underwent the 1‐week BH_4_ loading test. Patient background characteristics for those who underwent the 24‐h and/or 1‐week BH_4_ loading tests are shown in Tables 1 and 2 below.

**TABLE 1:** Characteristics of patients who received the 24‐h BH_4_ loading test.

Age	8–19 days	20–29 days	30–59 days	2–11 months	> 1 year	*p*	SMD	Overall	Missing (%)
*N*	69	42	31	12	12			170	
Sex Male/female% (freq)	41.8/58.2 (28/39)	35.7/64.3 (15/27)	41.4/58.6 (12/17)	33.3/66.7 (4/8)	58.3/41.7 (7/5)	0.683	0.231	40.6/59.4 (67/98)	2.9
Baseline blood test values (median [IQR])									
Phe (μM)	2154 [1473.6, 2435.4]	2050.8 [878.4, 2432.4]	441.6 [284.7, 881.4]	744 [181.5, 1126.5]	948.3 [575.55, 1387.65]	< 0.001	0.914	1628.40 [656.85, 2315.7]	1.2
Neopterin (nM)	151.58 [98.58, 214.05]	116.49 [78.62, 184.69]	51.45 [32.32, 77.88]	39.00 [30.58, 74.78]	39.86 [24.80, 65.97]	< 0.001	0.882	105.38 [55.19, 179.05]	1.2
Biopterin (nM)	59.45 [43.34, 83.70]	58.94 [37.06, 93.69]	28.46 [17.88, 70.84]	39.66 [25.54, 55.75]	78.06 [42.20, 103.81]	0.009	0.425	55.28 [29.83, 83.90]	1.2
N/B ratio	2.49 [1.67, 3.40]	2.21 [1.49, 2.79]	1.39 [1.11, 2.05]	1.15 [0.71, 2.22]	0.66 [0.51, 0.86]	< 0.001	0.7	2.00 [1.20, 2.90]	1.2

*Note:* Four patients had unknown age. All baseline blood test values were measured as plasma concentrations.

Abbreviations: N/B ratio, neopterin/biopterin ratio; Phe, phenylalanine; SMD, standardized mean difference.

**TABLE 2:** Characteristics of patients who received the 1‐week BH_4_ loading test.

Age	< 1 year	1–5 years	6–11 years	12–19 years	> 20 years	*p*	SMD	Overall	Missing (%)
*N*	8	28	18	13	50			117	
Sex Male/female% (freq)	37.5/62.5 (3/5)	17.9/82.1 (5/23)	33.3/66.7 (6/12)	46.2/53.8 (6/7)	32.0/68.0 (16/34)	0.42	0.274	30.8/69.2 (36/81)	0
Baseline blood test values (median [IQR])									
Phe (μM)	1510.2 [1061.25, 1753.5]	501.3 [349.89, 1137.9]	1093.8 [637.8, 1585.2]	1363.8 [1262.4, 1624.8]	1146 [715.8, 1513.8]	0.001	0.667	1135.8 [589.2, 1504.8]	0.9
Neopterin (nM)	26.38 [22.70, 32.08]	21.35 [13.13, 40.01]	20.89 [18.21, 28.69]	21.07 [11.84, 24.40]	18.42 [13.67, 22.43]	0.073	0.498	20.24 [14.12, 26.41]	1.7
Biopterin (nM)	32.92 [24.70, 45.65]	22.66 [14.18, 50.46]	40.50 [29.43, 57.25]	45.76 [26.58, 60.06]	36.46 [27.81, 52.83]	0.211	0.183	36.07 [20.54, 55.64]	1.7
N/B ratio	0.72 [0.57, 0.98]	0.80 [0.52, 1.47]	0.54 [0.40, 0.88]	0.46 [0.42, 0.53]	0.45 [0.38, 0.64]	< 0.001	0.649	0.52 [0.41, 0.80]	1.7

*Note:* All baseline blood test values were measured as plasma concentrations.

Abbreviations: N/B ratio, neopterin/biopterin ratio; Phe, phenylalanine; SMD, standardized mean difference.

### 3.1 Characteristics of the Patients Who Underwent the 24‐h BH_4_ Loading Test

According to the Japanese guidelines, the 24‐h BH_4_ loading test is performed shortly after birth, at the time when elevated Phe levels are identified by newborn screening [14]. Consequently, 66.9% of patients were younger than 1 month of age, and 85.5% were younger than 2 months (median age [IQR]: 22 days [16, 35.75]). Baseline Phe concentrations were high in those tested before 1 month of age (median 2121.6 μM [IQR: 1082.40, 2434.65]), whereas patients aged 30–59 days had lower levels (median 441.6 μM [IQR: 284.7, 881.4]). For those aged 2 months to adulthood, the median Phe level was 912.00 μM (IQR: 421.80, 1285.50). There were statistically significant differences in Phe levels among the age groups. Baseline biopterin levels also showed differences among age groups, consistent with those observed for Phe. In contrast, baseline total neopterin concentrations were elevated in the early neonatal period and decreased rapidly thereafter.

### 3.2 Relationship Between Biopterin Peak Value and Age in the 24‐h BH_4_ Loading Test

**FIGURE 2:**
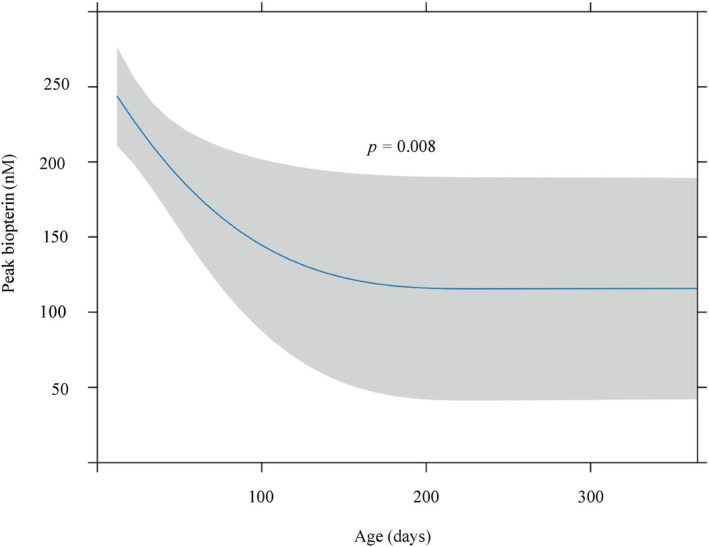
Relationship between biopterin peak value and age at time of the 24‐h BH_4_ loading test. Predictions (95% CI) from an ordinary least‐squares model of peak total biopterin with age (days), modeled by restricted cubic splines (3 knots) and adjusted for sex, baseline phenylalanine, baseline total neopterin, and baseline total biopterin. *p*‐values were obtained from two‐sided partial *F*‐tests (ANOVA/Wald) for the overall age effect.

Based on the hypothesis that the bioavailability of BH_4_ changes with age, we first analyzed the relationship between blood BH_4_ concentrations and age (Figure 2). In the single‐dose loading test, most patients were younger than 2 months of age (median [IQR]: 22 days [16, 35.75]). A negative correlation was observed between age in days and the peak total biopterin concentration following oral BH_4_ administration, with higher levels in the early neonatal period and a gradual decline with increasing age (*p* = 0.008).

In the 24‐h BH_4_ loading test, age‐stratified analyses included 110 patients aged < 30 days and 54 aged ≥ 30 days, after excluding cases with missing data (age, *n* = 4; baseline and/or peak biopterin, *n* = 2). In the patients aged < 30 days, day‐of‐age was negatively associated with the peak (*β* = −7.09 nM/day; 95% CI −12.6 to −1.60; *p* = 0.0119; Benjamini–Hochberg‐adjusted *q* = 0.024). The Breusch–Pagan test indicated heteroscedasticity (*p* = 0.001); using HC3 robust SEs, the association remained nominal (*p* = 0.0169; Benjamini–Hochberg‐adjusted *q* = 0.034). In the patients aged ≥ 30 days, no material association was observed (*β* = −0.0069 nM/day; 95% CI −0.0152 to 0.0015; *p* = 0.104; Benjamini–Hochberg‐adjusted *q* = 0.104; HC3 *p* = 0.059; Benjamini–Hochberg‐adjusted *q* = 0.059). An interaction model (age × < 30/≥ 30) confirmed that slopes differed across the 30‐day boundary (interaction *p* = 0.012; HC3 *p* = 0.029). Taken together, peak biopterin decreases with increasing day‐of‐age during the neonatal period (< 30 days), but is essentially flat thereafter.

### 3.3 Characteristics of the Patients Who Underwent the 1‐Week BH_4_ Loading Test

In Japan, there is no strict definition of the appropriate age for the 1‐week loading test, which is determined at the discretion of the attending physician [14]. As a result, the youngest patient in the 1‐week test was 5 months old, and the study population included a wide range of individuals from infancy to adulthood (median [IQR]: 14.77 years [4.97, 29.91]; number of patients aged < 1 year: 8, 1–5 years: 28, 6–11 years: 18, 12–19 years: 13, > 20 years: 50). Baseline Phe concentrations were lowest in the 1–5‐year age group (< 1 year: median 1510.2 μM [IQR: 1061.25–1753.5]; 1–5 years: 501.3 μM [IQR: 349.89–1137.9]; > 6 years: 1198.8 μM [IQR: 830.55–1527.9]). In patients younger than 1 year, total biopterin levels remained relatively low despite elevated baseline Phe concentrations (median: 32.92 nM [IQR: 24.70–45.65]). In contrast, among patients aged ≥ 1 year, the distribution of total biopterin levels showed a trend similar to that of baseline Phe concentrations (1–5 years: median 22.66 nM [IQR: 14.18–50.46]; 6–11 years: 40.50 nM [29.43–57.25]; 12–19 years: 45.76 nM [26.58–60.06]; ≥ 20 years: 36.46 nM [27.81–52.83]). Baseline total neopterin levels remained consistent across age groups, with an overall median of 20.24 nM (IQR: 14.12–26.41).

### 3.4 Relationship Between Peak Value of Biopterin and Age in the 1‐Week BH_4_ Loading Test

**FIGURE 3:**
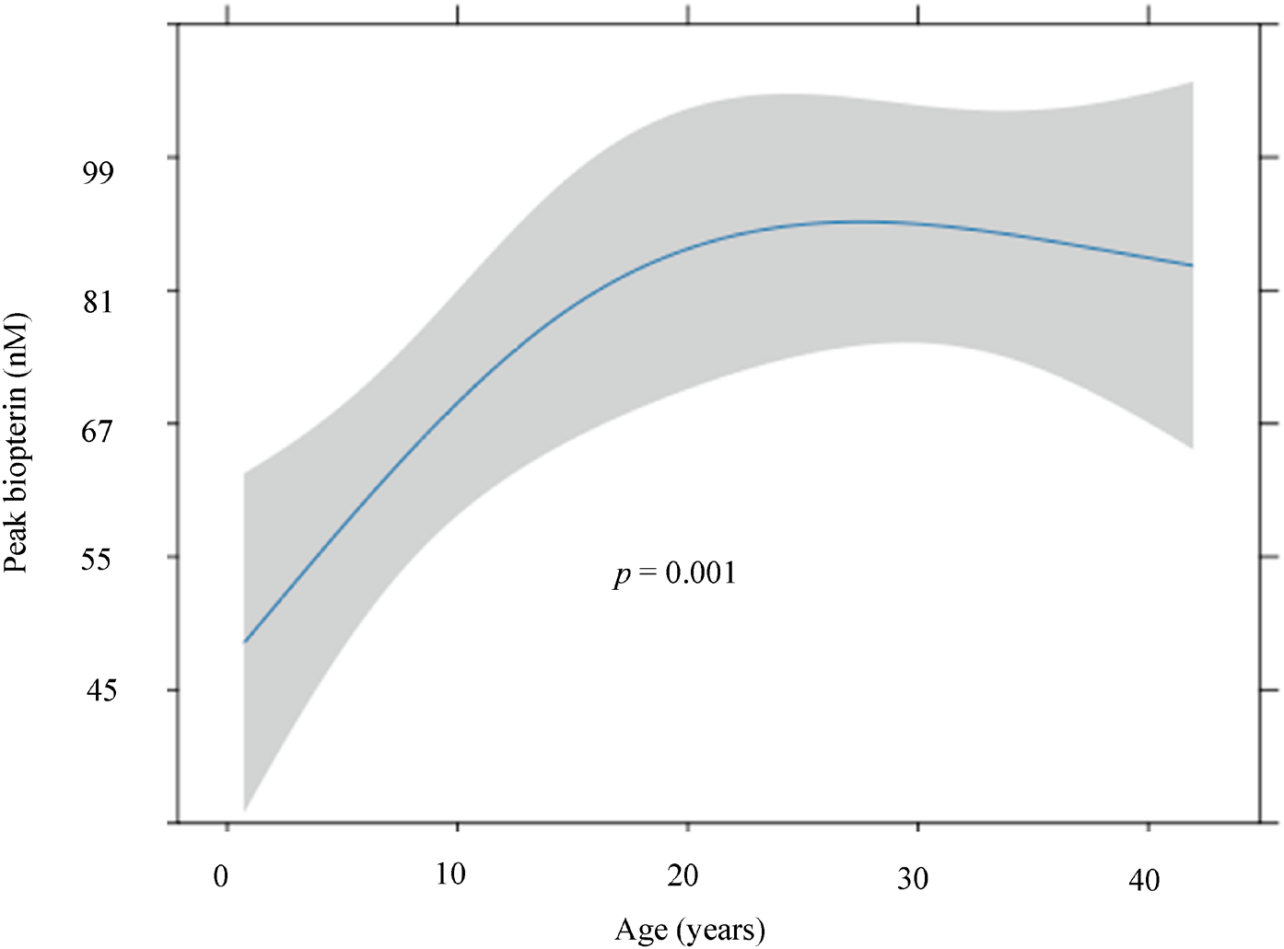
Relationship between biopterin peak value and age at time of the 1‐week BH_4_ loading test. Predictions (95% CI) from an ordinary least‐squares model of log (peak total biopterin) with age, modeled by restricted cubic splines (3 knots) and adjusted for sex, baseline phenylalanine, and baseline total neopterin/biopterin. *p*‐values were obtained from two‐sided partial *F*‐tests (ANOVA/Wald) for the overall age effect.

In the BH_4_ 1‐week loading test, biopterin peak levels increased significantly with age (*p* = 0.001) (Figure 3). In this test, the age‐stratified subgroups were *n* = 35 (< 6 years), *n* = 31 (6–19 years), and *n* = 48 (≥ 20 years); cases with unknown age were excluded (*n* = 3). For subjects aged 6–19 years, age showed a positive association with the biopterin peak value (*β* = +5.45 nM/year; 95% CI 1.78–9.12; *p* = 0.0051; Benjamini–Hochberg‐adjusted *q* = 0.015; adj. *R* ^2^ = 0.50). Heteroscedasticity was not detected (Breusch–Pagan *p* = 0.113). With HC3 robust SEs, the association remained directionally consistent but was weaker (*p* = 0.026; Benjamini–Hochberg‐adjusted *q* = 0.078). For subjects aged < 6 years, no association was observed (*β* = +0.35; 95% CI −9.47 to 10.2; *p* = 0.943; Benjamini–Hochberg‐adjusted *q* = 0.943; BP *p* = 0.765; HC3 *p* = 0.931). For subjects aged ≥ 20 years, again, no association was observed (*β* = −0.40; 95% CI −2.57 to 1.77; *p* = 0.709; Benjamini–Hochberg‐adjusted *q* = 0.943; BP *p* = 0.376; HC3 *p* = 0.691). In summary, age was positively associated with peak biopterin only during late childhood–adolescence (6–19 years); younger children and adults showed no material age trend.

### 3.5 Relationship Between BH_4_ ‐Responsiveness and Biopterin Peak Value in the 24‐h and 1‐Week BH_4_ Loading Tests

**FIGURE 4:**
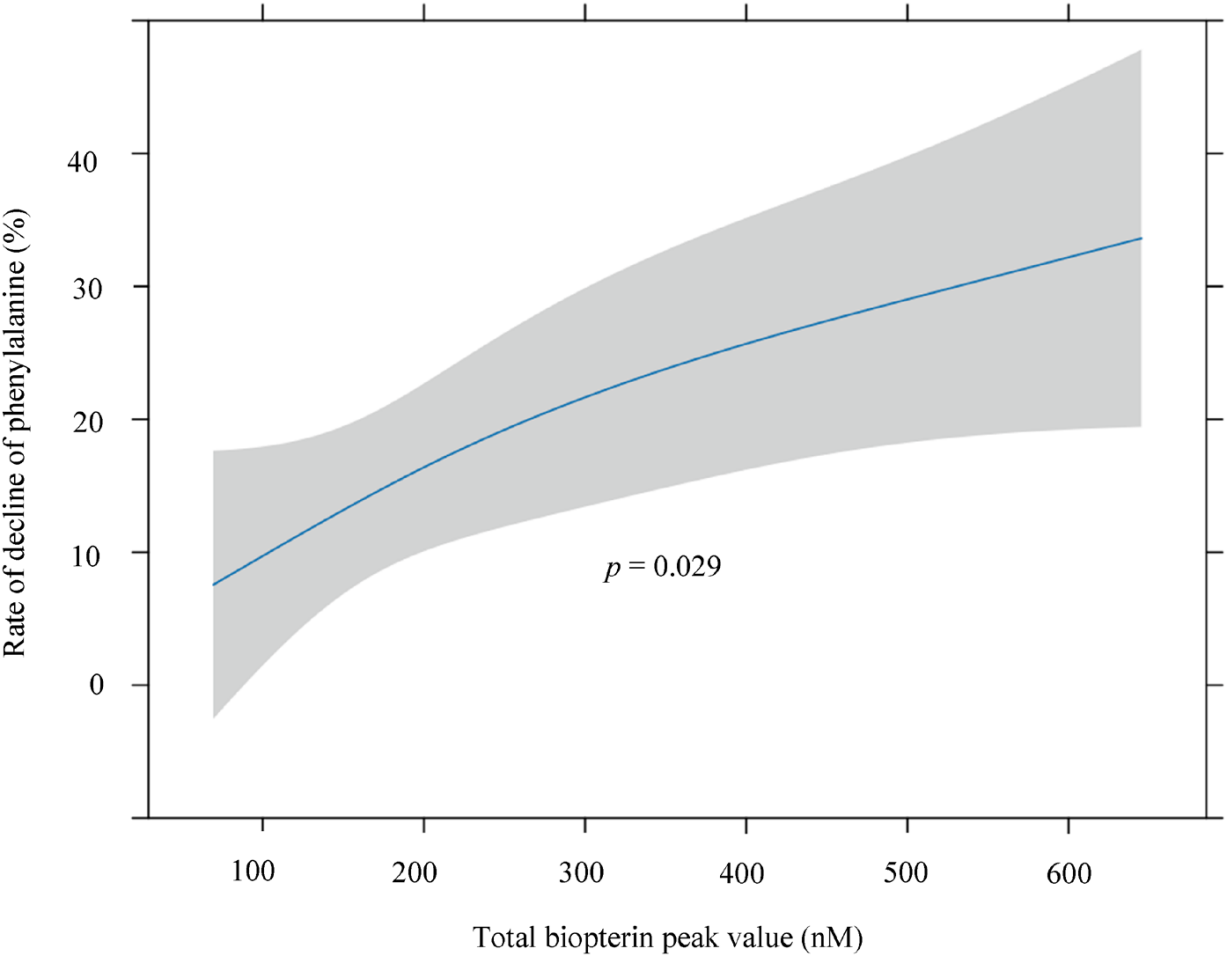
Relationship between rate of decline of phenylalanine (Phe) and biopterin peak value in the 24‐h BH_4_ loading test. The Phe reduction rate was calculated as the percentage decrease from baseline to the minimum Phe level observed during the 24‐h test. Predictions (95% CI) from an ordinary least‐squares model of Phe reduction rate with peak total biopterin, modeled by restricted cubic splines (3 knots) and adjusted for age (days), sex, baseline total neopterin, and baseline total biopterin. *p*‐values were obtained from two‐sided partial *F*‐tests (ANOVA/Wald) for the overall effect of peak total biopterin.

A non‐linear regression analysis, adjusted for age and sex, Phe and neopterin value, and baseline biopterin value, revealed a significant, positive correlation between peak total biopterin value and the rate of Phe reduction (Figure 4, *p* = 0.029).

### 3.6 Relationship Between Biopterin Value and Baseline Phe Value in the 24‐h BH_4_ Loading Tests

**FIGURE 5:**
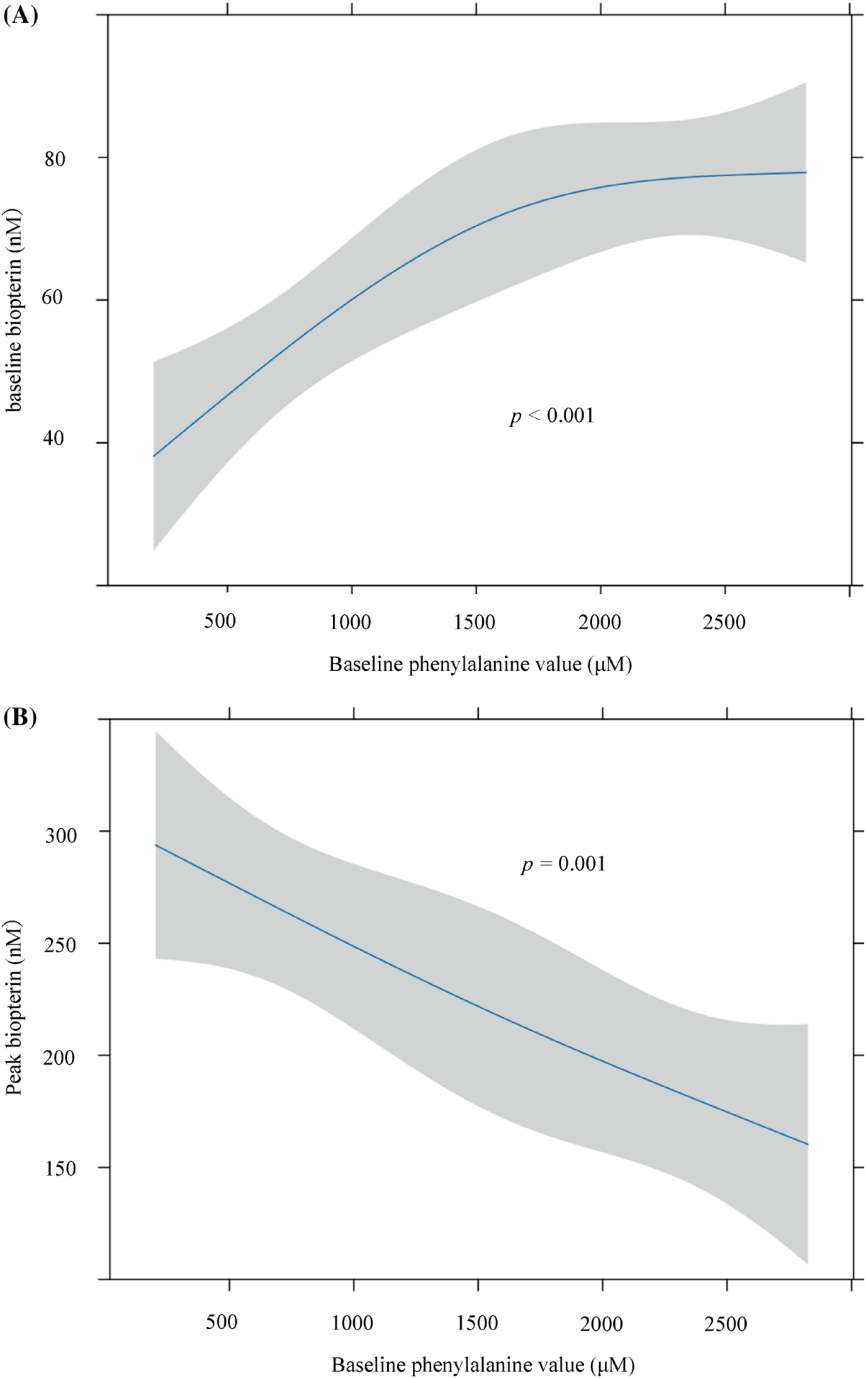
Relationship between baseline phenylalanine (Phe) and biopterin levels in the 24‐h BH_4_ loading test. (A) Relationship between baseline total biopterin and baseline Phe. Predictions (95% CI) from an ordinary least‐squares model of baseline total biopterin with baseline Phe, modeled by restricted cubic splines (3 knots). (B) Relationship between biopterin peak value and baseline Phe. Predictions (95% CI) from an ordinary least‐squares model of peak total biopterin with baseline Phe, modeled by restricted cubic splines (3 knots) and adjusted for age (days), sex, baseline total neopterin, and baseline total biopterin. *p*‐values shown in the panels were obtained from two‐sided partial *F*‐tests (ANOVA/Wald) for the overall effect of baseline Phe.

In the 24‐h loading test, higher baseline Phe concentrations were correlated with higher baseline biopterin levels (Figure 5A; *p* < 0.001). Furthermore, a non‐linear regression analysis, adjusted for age, sex, and baseline neopterin and biopterin values, revealed a significant negative correlation between peak biopterin value and baseline Phe level (Figure 5B, *p* = 0.001). Thus, a higher baseline Phe level was associated with a higher baseline biopterin level and a lower peak biopterin level (Figure 5A,B).

## 4 Discussion

This study presents the results of BH_4_ loading tests conducted at the only facility in Japan capable of performing pterin analysis for PKU. In Japan, newborn screening enables measurement of blood Phe concentrations in nearly all infants on Day 4 of life. For those with elevated Phe levels, early pterin analysis and BH_4_ loading tests are performed in accordance with national guidelines. Since the majority of these evaluations are centralized at our institution, the data presented here are considered representative of the broader Japanese PKU population.

Our analysis was based on the hypothesis that BH_4_ bioavailability changes with age and may affect the outcomes of the BH_4_ loading test. We found that BH_4_ bioavailability appears to be extremely high in the early neonatal period, followed by a rapid decline, and then a gradual increase through early childhood into adulthood. Moreover, peak total biopterin concentrations following BH_4_ administration were shown to be correlated with the degree of Phe reduction. Notably, in the 24‐h loading test, higher baseline Phe levels were associated with lower peak biopterin levels, suggesting that hyperphenylalaninemia itself may impair BH_4_ pharmacokinetics or metabolism. These findings suggest that both the age at which the BH_4_ loading test is performed and the baseline Phe levels may influence the test outcomes. Patients who undergo testing in the early neonatal period tend to have higher BH_4_ bioavailability and are therefore more likely to be diagnosed as BH_4_ responders. In contrast, patients with higher postnatal age or elevated baseline Phe levels may exhibit lower peak biopterin concentrations and a diminished Phe reduction response. Clinicians should recognize that BH_4_ loading tests performed in infants and preschool‐aged children (1 month–5 years)—in whom biopterin peaks tend to be lower—or in patients with high baseline Phe levels may yield suppressed biopterin peaks and lead to false‐negative results. Reassessment of BH_4_ responsiveness and age‐appropriate dose adjustment should be considered when necessary. These insights are important clinically for the diagnosis of PKU and the subsequent therapeutic decision‐making.

### 4.1 Association Between Age and Biopterin Peaks in the 24‐h Test

In the 24‐h BH_4_ loading test, peak total biopterin concentrations following BH_4_ administration were high in the early neonatal period and decreased sharply with increasing postnatal age (Figure 2), making this the first report to demonstrate age‐related changes in peak biopterin concentrations following BH_4_ administration in human neonates. In the age‐stratified subgroup analyses, this trend was significant within the neonatal period. Our previous case report [11] described repeated 24‐h BH_4_ loading tests in two patients with BH_4_‐responsive hyperphenylalaninemia. In the first case, peak blood biopterin concentrations following oral administration of 10 mg/kg BH_4_ were 612, 297, and 178 nM at 30 days, 55 days, and 19 months of age, respectively. In the second case, the peak values were 747 nM at Day 20 and 327 nM at Day 55. These findings indicate that BH_4_ bioavailability was higher at younger ages, and that peak biopterin levels declined markedly after the neonatal period. Animal studies have also shown that younger rats exhibit higher BH_4_ absorption, likely due to the immaturity of the intestinal mucosa in neonatal animals, which leads to insufficient mucosal barrier function and allows BH_4_—despite its very low lipid solubility—to pass through the intestinal epithelium [15]. Although pharmacokinetic parameters following oral sapropterin administration have been reported in children and adults [7, 16, 17, 18], direct data regarding the specific mechanisms of age‐dependent absorption changes in human neonates remain limited. Furthermore, considering that renal function matures rapidly in the early postnatal period, leading to improved clearance of renally excreted drugs [19], it is also possible that this rapid renal development contributes to the extremely high biopterin peaks observed during the neonatal period, independent of absorption alone.

### 4.2 Association Between Age and Biopterin Peaks in the 1‐Week Test

In the population ranging from 5 months of age to adulthood that underwent the 1‐week BH_4_ loading test, peak total biopterin levels increased during school age through adolescence (6–19 years), with no clear age trend in adults (≥ 20 years) (Figure 3; age‐stratified analyses). The 1‐week loading test results suggest that the association between age and BH_4_ bioavailability is not uniform across an individual's lifespan. In the age‐stratified subgroup analyses, the significant increase in the 6–19‐year group suggests that during school age through adolescence—when body weight increases more rapidly—the peak blood biopterin after oral BH_4_ at 20 mg/kg may rise substantially. Similarly, in the phase IIIb SPARK trial by Muntau et al., a population pharmacokinetic model demonstrated that lower‐weight subjects exhibited lower plasma biopterin concentrations after receiving 10 mg/kg of sapropterin dihydrochloride. The findings of our study are consistent with this trend, indicating a weight‐dependent pharmacokinetic profile for BH_4_ [7, 16]. This suggests that in younger children with lower body weight, blood concentrations tend to be lower, and dosage adjustments may be appropriate.

Furthermore, we must consider the potential impact of age‐related comorbidities on BH_4_ metabolism. Recent studies have highlighted that adult patients with PKU exhibit a higher prevalence of comorbidities, including obesity, hypertension, and cardiovascular risk factors, compared to the general population [20, 21, 22]. These conditions are pathophysiologically characterized by chronic systemic oxidative stress and endothelial dysfunction, leading to the “uncoupling” of endothelial nitric oxide synthase (eNOS) [23].

Under conditions of high oxidative stress, particularly in the presence of peroxynitrite, BH_4_ is not only oxidized to dihydrobiopterin (BH2) but can also undergo irreversible side‐chain cleavage to form pterin and xanthopterin [24, 25]. It is important to note that the biopterin assay method used in this study measures “total biopterin” (the sum of BH_4_, BH2, and biopterin) but does not detect side‐chain cleavage products such as pterin. Therefore, the lack of an increase or the relative suppression of peak biopterin levels observed in our adult cohort may reflect an accelerated metabolic loss of BH_4_ into undetectable degradation products, driven by the “oxidative sink” associated with adult comorbidities.

### 4.3 Association Between Biopterin Peaks and Phe Reduction

In the present study, a statistically significant correlation was observed between peak total biopterin levels and the rate of Phe reduction in Japanese PKU patients (Figure 4, *p* = 0.029). These findings suggest that if blood biopterin levels are low during the BH_4_ loading test, particularly in patients with genotypes whose BH_4_ responsiveness has not been fully characterized, there is a risk that true responsiveness may be overlooked. In human hepatocytes, BH_4_ is physiologically maintained at a molar concentration equivalent to that of PAH protein (approx. 10 μM) [12, 26]. However, since the K_m_ of BH_4_ for wild‐type human PAH is approximately 24 μM, PAH enzyme activity remains relatively low under physiological BH_4_ concentrations. Under conditions of elevated Phe levels, an increase in BH_4_ concentration is required for human PAH to achieve its peak enzymatic activity [27, 28].

Regarding this point, Gundorova et al. recently proposed the concept of three‐dimensional “PAH activity landscapes” through in vitro functional phenotyping using automated high‐throughput screening of PAH variants expressed in cell culture models. According to their report, the optimal Phe and BH_4_ concentrations required to maximize residual activity differ for each variant. Specifically, genotypes characterized by “right‐shifted” landscapes—represented by variants such as p.Glu390Gly, p.Asp415Asn, p.Ile65Thr, and p.Arg261Gln—require high Phe concentrations and sufficient BH_4_ levels to exhibit enzymatic activity [29]. Consequently, if BH_4_ bioavailability is insufficient, as observed in the infants in our study, or if the test is performed when Phe levels are relatively low, the therapeutic potential of BH_4_ in these “conditional” responders may be underestimated.

Our clinical experience with Japanese PKU patients provides concrete examples of these landscape concepts. For instance, the *PAH* p.Arg241Cys genotype represents a robustly responsive variant where many patients can discontinue dietary restrictions with standard BH_4_ doses. In contrast, variants such as *PAH* p.Ser70del are likely to align with “right‐shifted” landscapes, requiring much higher BH_4_ concentrations to elicit a therapeutic effect. In such cases, standard doses of 10–20 mg/kg BH_4_ may have limited efficacy, whereas sepiapterin—which achieves higher intracellular BH_4_ concentrations—may be required to “reach” the active portion of the landscape [30, 31].

Furthermore, our previous case report provides concrete examples of these landscape concepts. In the patient with the *PAH* p.Arg241Cys/Arg111X genotype, at Day 20, a baseline Phe of 1180 μM and a biopterin peak of 747 nM resulted in a Phe reduction rate of 39%. Even at Day 55, when the biopterin peak was 327 nM (baseline Phe 848 μM), the Phe reduction rate showed only a minimal decline to 32%. In contrast, the patient with the *PAH* p.P407S/R158W genotype appears to possess a landscape more sensitive to BH_4_ and Phe concentrations. At Day 30, with a baseline Phe of 638 μM and a biopterin peak of 612 nM, the Phe reduction rate was 54%. However, at Day 55, despite a similar baseline Phe (593 μM), the decline in the biopterin peak to 297 nM caused the Phe reduction rate to plummet to 16%. By 19 months of age, with a baseline Phe of 263 μM and peak biopterin of 178 nM, the Phe reduction rate was very low at 4% [11]. Similarly, Zurflüh et al. reported a patient (*PAH* p.Ala403Val/Ser411Ter) who showed an insufficient response in the initial test but a favorable Phe reduction when a subsequent test performed a few weeks later achieved a higher biopterin peak [32], further emphasizing the threshold effect within these activity landscapes.

For the first time, this study demonstrated in a human PKU population that cases with a well‐elevated blood biopterin concentration during the BH_4_ loading test tended to show a favorable reduction in Phe levels. These findings suggest that re‐evaluation may be warranted in cases where blood biopterin levels fail to rise adequately during the BH_4_ loading test, especially when a genotype suspected of having high Phe and BH_4_ requirements is involved.

### 4.4 Impact of Baseline Phe on Biopterin Peaks

In the 24‐h loading test, which primarily involved infants younger than 2 months of age, higher baseline Phe concentrations were associated with higher baseline biopterin levels because high Phe stimulates GTPCH activity (Figure 5A). Interestingly, even though higher baseline Phe resulted in higher baseline BH_4_, peak biopterin levels were lower (Figure 5B).

Two potential mechanisms may explain this observation. Although the effects of elevated Phe levels on BH_4_ metabolism are not fully understood, the first potential mechanism is that Phe‐induced oxidative stress [33] accelerates the degradation of BH_4_. Elevated Phe levels are known to stimulate GTPCH (GTP cyclohydrolase I) activity, thereby promoting BH_4_ synthesis; however, they concurrently induce oxidative stress accompanied by increased reactive oxygen species (ROS) and NADPH oxidase (Nox) upregulation [33, 34]. As discussed in Section 4.2, oxidative stress causes the irreversible inactivation of BH_4_ via side‐chain cleavage. Consequently, under high‐Phe conditions, it is likely that both BH_4_ synthesis and its degradation are accelerated. In this state, orally administered BH_4_ would be rapidly degraded, potentially explaining the observation of low peak BH_4_ levels despite elevated baseline BH_4_ values.

A second possible mechanism is that certain genotypes associated with high Phe levels may intrinsically exhibit a reduced capacity to achieve peak biopterin concentrations. In PKU model mice, a phenomenon referred to as “secondary BH_4_ deficiency” has been reported [35], in which misfolded and unstable mutant PAH proteins accumulate in the liver and bind BH_4_ to form inactive complexes, thereby reducing the amount of free BH_4_. The affinity of mutant PAH proteins for BH_4_ appears to vary depending on the genotype and is particularly strong in mice homozygous or heterozygous for the PAH p.Val106Ala variant [35]. This mechanism suggests that, in some PKU patients with specific genotypes, orally administered BH_4_ may be preferentially taken up into tissues and bound to PAH, resulting in a smaller‐than‐expected increase in circulating BH_4_ levels [35].

These findings align with our observation that patients with higher baseline Phe levels tended to show smaller increases in blood biopterin concentrations.

### 4.5 Limitations

In this study, baseline Phe and biopterin levels varied significantly across age groups, suggesting that differences in background factors may have influenced the results. In the 24‐h loading test, many subjects were untreated, and Phe levels were particularly high in those under 30 days of age (median: 2121.6 μM), suggesting that more severe cases were selectively tested early. In contrast, the 1‐week test involved patients under treatment, and because patients were instructed to follow a regular diet through self‐management before and during the test, variability in treatment adherence and dietary intake likely affected Phe levels. Indeed, in this study, a decrease in Phe levels during early childhood and an increase during adolescence were observed, consistent with reduced treatment adherence with age [2]. Moreover, fluctuations in Phe may influence biopterin levels via GTPCH activity [36, 37]. Given that these factors are intermingled in the baseline values across both tests, baseline Phe and biopterin levels were included as covariates in all the analyses.

In the BH_4_ 1‐week loading test protocol, blood samples were collected on Days 4 and 7; however, the timing of sampling relative to BH_4_ administration was not standardized. Given that biopterin levels exhibit diurnal variation even at steady state, the random timing of blood collection may have introduced variability unrelated to age. Nevertheless, this study included a relatively large sample size and demonstrated statistically significant age‐related differences across a wide age range. While these variations are unlikely to have major clinical implications, it is important to note that drug blood concentrations may be lower in small infants due to their smaller body size.

## 5 Conclusion

We reported the characteristics of patients in Japan who underwent 24‐h or 1‐week BH_4_ loading tests. Following oral BH_4_ administration, peak total biopterin levels were highest in the early neonatal period in the 24‐h test and then declined rapidly, whereas in the 1‐week test, levels increased during school age through adolescence (6–19 years), with no clear age trend in adults (≥ 20 years). In the 24‐h BH_4_ loading test, a positive correlation was observed between total biopterin peak levels and the Phe decline rate. Conversely, cases with high baseline Phe levels showed lower biopterin peaks after BH_4_ administration. Therefore, when BH_4_ loading tests are performed in infants and preschool‐aged children (1 month–5 years)—in whom biopterin peaks tend to be lower—or in patients with high baseline Phe levels, the biopterin peak may not rise sufficiently, increasing the risk of a false‐negative assessment of BH_4_ responsiveness. In such cases, re‐evaluation of BH_4_ responsiveness or dose adjustment according to age should be considered.

## Author Contributions

Kana Kitayama (first author) conceived and designed the study, collected the data, performed the statistical analysis, and drafted the manuscript. Tomoko Sakaguchi and Noriko Nakano (clinical laboratory technologists) were responsible for the pterin analysis and genetic testing. Daijiro Kabata made a significant contribution to the planning and execution of the statistical analysis and provided essential methodological advice. Takashi Hamazaki and Haruo Shintaku provided overall supervision of the project, contributed substantially to the study design and interpretation of data, assisted in data collection, and offered critical guidance throughout the manuscript preparation. All authors critically revised the manuscript for important intellectual content, approved the final version, and agreed to be accountable for all aspects of the work.

## Funding

This research was supported by the Japan Agency for Medical Research and Development under grant number 25ek0109636h0003 awarded to T.H.

## Ethics Statement

This retrospective study was approved by the Ethics Committee of Osaka Metropolitan University Graduate School of Medicine (approval no. 2023‐110, December 11, 2023).

## Consent

Written informed consent was not obtained because this was a non‐invasive observational study using existing clinical data. In accordance with Japanese guidelines, an opt‐out procedure was implemented via the department website.

## Conflicts of Interest

Takashi Hamazaki has participated in advisory boards for BioMarin and PTC Therapeutics Inc., and received clinical trial support from BioMarin, PTC Therapeutics Inc., and Otsuka. Takashi Hamazaki has also received honoraria for lectures from Daiichi Sankyo.

## Data Availability

Research data are not shared.
